# Book Reviews

**Published:** 1831-04

**Authors:** 


### CRITICAL ANALYSES.

Quæ laudanda forent, et quæ culpanda, vicissim

Illa, prius, cretâ; mox hæc, carbone, *notamus*.—PERSIUS.

*A Treatise on Pathological Anatomy*. By G. ANDRAL, Professor to the Faculty of Medicine of Paris, &c. *Translated from the French*, by RICHARD TOWNSEND, A.B. M.D. M.R.I.A. and WILLIAM WEST, A.M. M.D. M.R.I.A. Vol. II. —8vo. pp. 830. Dublin: Hodges and Smith. London: Longman and Co. 1831.

(Continued from our last Number.) p. 258.

WE now arrive at another division of this interesting work, namely, an account of those diseases of the alimentary canal affecting parts above the diaphragm; and first, of "*Lesions of the Mouth and Pharynx*." M. ANDRAL has nothing particular to offer concerning the various degrees of hyperæmia, softening, and induration, that may be presented by the stomato-pharyngean mucous membrane, as they resemble those already described, when treating of the stomach and intestines; but, among other peculiar alterations which happen in this membrane, is that kind of affection described by Bretonneau, under the name of *diphtheritis*, which, from the description given of it, we take to be another term for the *aphtha* of most writers.

The tonsils are subject to several morbid alterations.

"The seat of these alterations is sometimes in the cellular tissue situated between the follicles, which may either be simply congested, secrete pus, or become indurated and enlarged, thus producing the so-called scirrhous state of the tonsils. At other times it is the follicles themselves, as well as the lacunæ into which they open, that are altered either in the texture of their parietes, or in the fluid contained in their cavities. Their parietes, like those of every other follicle, are found, according to the case, either in a state of simple hyperæmia, or of hypertrophy, induration, or softening. The fluid naturally secreted by them may be modified in its quality, so as to become either pus, or a concrete friable substance, like tubercle, or one still more solid, of sufficient consistence to be termed a calculous concretion, and varying in size from the bulk of a grain of millet to that of a kidney bean. These various morbid secretions seem to be contained, sometimes in a single lacuna considerably enlarged, and sometimes in a cavity formed by the accidental union of several. These facts are not merely interesting from the situation of the parts affected, but also from the light they throw on the nature of the alterations of texture of other portions of the mucous membranes, in which accidental cavities containing pus, tuberculous matter, or calculous concretions, seem likewise to be merely follicles altered in the structure of their parietes, and in the qualities of their secretion." (P. 272.)

Hypertrophy of the tonsils is by no means uncommon, and may exist without induration of the parts.

Gangrene of the mouth is thought, by Andral, to be less common than is usually supposed. It is more frequently a disease of childhood, and is scarcely ever met with except among the poorer classes, whose habitations and food are unwholesome, or in such as have a highly scrofulous constitution, or shew symptoms of scurvy. This affection may be confined to the mucous membrane only, which it destroys to a greater or less extent; it may extend its ravages to the subjacent tissues, and proceed to the periosteum and bones.

"When the disease arrives thus far, the teeth already grown fall out, the roots of the second set are also destroyed, and, if the patient recover, he probably remains toothless the rest of his life. At the same time, the child grows remarkably pale; its cheeks become

œdematous, its whole body wastes rapidly away, the pulse becomes small and wretchedly feeble, the skin cold, and, if the progress of the gangrene be not arrested, death speedily follows. Some authors see, in all this train of symptoms, nothing but stomatitis terminating in gangrene. But, if it be true that this gangrene occurs chiefly under certain morbid conditions of the innervation and sanguification, and if, moreover, its appearance is not preceded by any perceptible inflammatory reaction, should we not, in this case also, consider the affection of the mouth to be connected with a morbid state that is not confined to the part where the gangrene appears? In like manner, in those cases where the use of rye affected with ergot, as an article of food, is followed by gangrene of the inferior extremities, the cause of the gangrene is not to be found in those extremities." (P. 275.)

*Lesions of the Œsophagus.* The epithelium, which covers the mucous membrane of the œsophagus, is sometimes eroded, softened, and destroyed, especially in the inferior part of the œsophagus. In some cases, M. Andral has found the epithelium remarkably thickened. The mucous membrane of the œsophagus presents the same alterations as in the other parts of the alimentary canal. Its parietes sometimes become attenuated and softened; one of the consequences of which is spontaneous perforation, which offers the same anatomical characters as that of the stomach. Cases of gangrene of the œsophagus have been described, but many, if not all of them, are, in M. A.'s opinion, merely cases of pultaceous softening of the part. The same lesions of secretion may occur in the œsophagus as in the other portions of the alimentary canal.

The second chapter we pass over: it contains an account of various congenital malformations of the upper part of the alimentary canal.

Circulatory Apparatus; and first of diseases of the heart, in which hyperæmia, or vascular congestion, plays a very secondary part, when compared with the important character it assumes in diseases of the lungs and alimentary canal. The lesions of nutrition and secretion are also comparatively unimportant, unless when they proceed so far as to produce an alteration in the dimensions of the heart, or a disproportion either in the thickness of its parietes, or in the capacity of its cavities, and thereby create an obstacle to the entrance or exit of the blood.

*Lesions of Circulation.* It is, in many cases, difficult to decide whether the various shades of red observable in the substance of the heart, or on its internal surface, should be regarded as the marks of a hyperæmia formed during life, or as an alteration produced after death, and altogether dependent on physical causes. In fact, the heart is one of the organs which evince the greatest aptitude to these post-mortem colorations.

"If we open an animal shortly after it is killed, and examine the heart, we find its internal surface pale, and its substance but faintly coloured; but if the same heart be exposed to the air and sun for a few hours, its internal surface changes to a bright scarlet, and its muscular structure assumes a deep red colour. When the examination of the body is deferred, in hot or damp weather, until thirty hours after death, the heart is invariably found red: in some cases the redness is confined to its internal surface, which then appears as if dyed by some red colouring matter, either uniformly, or in insolated patches; in other instances, the red colour extends through its parietes, to which it imparts a dark livid hue, which is occasionally accompanied by the appearance of ecchymoses on its external surface.

"This post-mortem colouring of the heart is not always found to the same extent even in those bodies that are opened under similar circumstances. *Cæteris paribus,* it is most marked in those individuals who at the time of their death have a large supply of blood in the system; some bodies too have a much greater tendency to putrefaction than others, and in them the red colour of the heart is always exceedingly marked." (P. 297.)

The morbid anatomist must, therefore, be cautious how he attributes all red appearances in the heart to inflammation of that organ, as the greater proportion of them are to be referred to post-mortem alterations. But it is true that these appearances are occasionally produced during life, and are connected with a true state of active hyperæmia, i. e. inflammation.

*Anæmia* of the heart has been as yet but little attended to. It not unfrequently succeeds to a state of hypertrophy and congestion. There are various lesions of nutrition that impede the free circulation of the blood through the heart. The nutrition of the heart may be so modified as to produce various alterations in the dimensions of its cavities, or in the thickness of its parietes. Increased thickness of the heart's parietes is generally designated by the term *hypertrophy:* of this alteration in the nutrition of the heart, there are various species, all of which M. Andral describes. "The parietes of the heart, when affected with hypertrophy, may retain their natural consistence, which is by far the commonest case; or may be more or less indurated, which is rare; or may be softened, which is still more uncommon." Hypertrophy of the parietes of the heart may accompany different conditions of its cavities, and each of these varieties is characterised by a peculiar train of symptoms.

*Atrophy* of the walls of the heart is less common than hypertrophy.

"Atrophy of the heart sometimes proceeds so far that its parietes are reduced to mere membranes, in which scarcely a trace of muscular fibre is distinguishable. In such cases, the fat which naturally exists around the heart is generally much increased, and its secretion becomes more active in proportion as the muscular fibres disappear.

"In general, atrophy of the heart comes on without any assignable cause. As anæmia of this organ may succeed to its hyperæmia, so its atrophy may succeed to a preceding hypertrophy. Laennec records the case of a woman, fifty years old, who for twelve months had constantly presented all the symptoms of organic disease of the heart, from which she was subsequently relieved by Valsalva's method of treatment. Two years after her recovery, she was seized with cholera morbus, and died. On dissection her heart was found remarkably small, scarcely exceeding in size the heart of a child of twelve years of age. Its external surface bore an exact resemblance to a wrinkled apple, the wrinkles being generally directed from the apex towards the base." (P. 307.)

In several diseases, in which persons die much emaciated, the heart is found, on dissection, to have participated in the general atrophy of the muscular system. This is not always the case; for, in many phthisical patients, who die in an extreme state of marasmus, the heart retains its natural volume; or, if its size appears diminished, its diminution is apparent, and depends on collapse of the parietes, in consequence of their containing little or no blood. Mechanical obstacles to the circulation may reside either in the orifices of the heart, in the arteries, or in the capillaries. The orifices of the heart are occasionally so narrowed, either by congenital malformation or by disease, as to prevent the free passage of the blood. Instead of this simple narrowing of the apertures, the obstacle to the circulation may be produced by various organic lesions of the valves which surround them.

"The orifices of the heart may continue for a long time in a state of disease, without producing habitually any appreciable derangement of its action. But, in such cases, any effort, or violent exercise, and mental exertion, or excess of any kind, in short, any thing tending to accelerate the circulation, seldom fails to produce palpitations and dyspnœa." (P. 314.)

M. Andral points out certain malformations of the aorta, which may more or less impede the circulation of the blood. How far obstacles seated in the general capillary system are capable of affecting the circulation in the heart, we know not; but, in M. A.'s opinion, there are few points in pathology better established than that any impediment to the capillary circulation of the lungs may cause hypertrophy and dilatation of the heart. "In fact, the lung in which the circulation of the blood is impeded, or otherwise retarded, is to the right side of the heart, what the liver, when gorged or *obstructed*, is to the vena portæ." From the numerous researches which the author has himself made on the subject, he is quite convinced that congestion of the bronchial membrane, in chronic catarrhs, is a frequent cause of the diseases of the heart, with which they are so constantly complicated.

"In such cases, the difficulty of breathing is often long antecedent to any local symptom of organic disease of the heart: and if, in phthisical persons, disease of the heart is comparatively a rare affection, although their pulmonary circulation is often so materially impeded, a sufficient explanation is afforded by the circumstance of the rapid diminution which takes place in the mass of their circulating fluid. Neither do we observe in them those long, distressing paroxysms of coughing, which occur in chronic catarrhs, and which by their repetition necessarily derange and impede the circulation in the pulmonary artery, and consequently in the heart itself." (P. 315.)

The action of the heart may be deranged without any alteration in its texture. The heart, which beats too violently or too rapidly, is not more necessarily altered in its organization than any other muscle which contracts irregularly, and independently of the will. The nervous system is the agent of these movements: it is, therefore, consistent with the soundest physiology to admit, in the case of the heart, as well as in that of the muscles of animal life, that a modification of the nervous influence may cause a modification of its contractions. But we know that the muscle, whose contractions are, for any length of time, rendered more frequent or more violent by the influence of the nervous system, in the end becomes hypertrophied; and why should not the same cause produce the same effect in the heart. Upon these grounds, M. Andral contends, that the term *nervous palpitation* is not, as has been said, a phrase by which we endeavour to conceal our ignorance, but the expression of a positive fact. We may find every variety of hypertrophy of the parietes of the heart, or of dilatation of its cavities, unaccompanied by the slightest evidence of any antecedent irritation of its surfaces, or the appearance of any obstacle whatever in the rest of the circulatory apparatus.

Different lesions of nutrition may exist, as induration or softening, that do not alter the dimensions of the heart. The first is to be carefully distinguished from hypertrophy; for, though generally combined, they are in reality different affections, and may exist independently of each other. The softening of the muscular tissue constitutes what is, strictly speaking, termed *softening of the heart.* This condition may be either general or partial.

"According to Laennec, we may expect to find the heart softened in those cases where dilatation of that organ has been accompanied by long and repeated attacks of suffocation; where the mortal struggle has been protracted for several weeks; and where the violet colour of the face and extremities has announced, long before death, the stagnation of the blood in the capillary system. I do not mean to deny that the heart may occasionally be found softened after this series of symptoms, but I can affirm, from my own experience, that it by no means necessarily follows. There is another assertion of Laennec's, which I think requires further confirmation before we can admit it as well established, namely, that the yellow softening of the heart is generally accompanied by a certain degree of cachexy." (P. 322.)

The heart may suffer different solutions of its continuity: ulceration is a rare affection. Rupture of the heart has been said to succeed to falls or violent efforts. M. Andral can scarcely conceive that strong mental emotions could produce this effect, unless the heart was predisposed to rupture by some preceding lesion. Perforations of the heart have been found accompanied by various conditions of the general structure of the organ. M. A. exhibited recently to the Academy of Medicine a heart, which had five perforations in its left ventricle; its muscular structure had no appearance of softening. It is stated that perforation of the heart is generally followed by sudden death, not from hemorrhage, for the pericardium limits its quantity. In several such cases, M. Andral found only a small clot of blood, not even sufficient to distend the investing membrane. He thinks it most probable that death is caused, in these instances, by the violent shock to the whole system, produced by the sudden derangement of the heart's functions.*

"Of the individuals who died under my care, in consequence of rupture of the heart, some had for a long time previously manifested the usual symptoms of organic disease of that organ; others had never betrayed any symptom of disease either of the heart or large vessels; and others, again, had complained occasionally of uneasiness or pain in the precordial region, unattended with any other morbid symptom.

* Although we cannot, at this moment, quote the authority, we perfectly remember to have seen it stated, that, in three horses struck dead by lightning, the heart of each was found to be ruptured.—Editor.

"Instead of ensuing, as it generally does, almost instantaneously, death in some cases does not take place for several hours, or even days, after the accident, in which case the perforation is found plugged up by a coagulum of fibrine. Is it possible that this coagulum could become the medium for forming a true cicatrix, as in wounds of arteries, and, consequently, that perforation of the heart is not necessarily mortal?" (P. 326.)

Rupture of the chordæ tendineæ, or carneæ columnæ, has frequently been found to exist. Various congenital malformations of the heart have been seen: the heart has been found wanting; it may be imperfectly or excessively developed; the direction and situation of this organ may be unnatural. It is curious that almost all the cases of acardia, hitherto observed, have been in fœtuses wanting the brain and spinal cord.

*Lesions of Secretion.* In the healthy state, the only secretions which take place in the heart are, the secretion of fat between its muscular fibres, and the insensible exhalation of serous fluid. Hence we have two orders of the lesions of secretion: a modification of the fatty exhalation, and a modification of the serous exhalation. When the general emaciation is considerable, the quantity of fat deposited round the heart is diminished, or totally disappears. Sometimes the adipose matter is excessive, and it then penetrates between the muscular fibres of the heart, which, in consequence, become pale and wasted.

"This has been called the fatty degeneration of the heart, though in reality the muscular fibres are not converted into fat, but are only less apparent than usual, in consequence of the excessive deposition of fat between them. In some cases, however, it would appear that the muscular fibre itself had undergone a fatty transformation, a matter capable of greasing paper, and the scalpel being found not only between the fibres, but infiltrating their texture. I have never myself seen this transformation of the muscular fibre into fat, but at the apex of the heart; neither has Laennec seen it except in the same situation, or in other very circumscribed spots. It is accompanied by a yellow tinge like that of decayed leaves." (P. 337.)

An accumulation of fat about the heart has been considered a cause of asthma and sudden death. The author admits the possibility of this fact, but he thinks proofs of it are yet required.

Lesions of the perspirable exhalation of the heart may occur in the parenchymatous substance of the organ, and on the surface of its cavities. The quantity of fluid which the cellular tissue of the heart exhales, is sometimes so much increased as to produce a serous infiltration, or true œdema of that viscus. M. Bouillaud has only seen it in cases of general dropsy, and attributes it to the same causes which give rise to the general affection. Instead of the perspirable fluid, we sometimes find separated from the blood certain morbid productions, both solid and fluid.

Ossification of the heart itself, or one of its component tissues, occasionally exists.

"It remains still to be explained why the ossiform degeneration is almost exclusively confined to the left side of the heart, although the structure and organization of both sides seem precisely the same. Another circumstance which we are equally at a loss to account for, is the frequency of this degeneration in old age, and its comparative rarity before the age of fifty or fifty-five. It is one of those striking examples, of which we possess so many, of the subordination of local affections to certain general modifications of the system." (P. 342.)

There are two sorts of abscesses of the heart: one produced by a morbid condition of the heart itself, the other occurring only in those cases where there has been a suppurative process going forward in some other part of the body, from which the pus is absorbed into the circulation, and subsequently deposited in different points of the system. M. Reynaud shewed the author a heart which had several collections of pus in the substance of its parietes, and a considerable quantity of the same fluid mixed with the blood in its cavities. The morbid productions known by the name of *tubercles, scirrhus,* and *encephaloid,* are found occasionally in the heart. As far as the experience of M. Andral extends, tubercles are never found in the heart, but when they exist at the same time in several other organs. Serous cysts and hydatids are sometimes observed in the heart.

"The only instance, I believe, on record, of these cysts being developed on the free surface of the lining membrane of the heart, is one which fell under my own observation. A small serous cyst, about the size of a nut, was attached to the internal surface of the right ventricle, near its auricular orifice, by a delicate pedicle, apparently of the same texture as the internal membrane." (P. 348.)

There are few lesions of the perspirable exhalation in the cavities of the heart. M. Andral knows of no other morbid productions in the cavities, except pus and false membranes, and he deems it very difficult, in many cases, to decide whether the pus so found is really the product of inflammation of the lining membrane of the heart, or not, as it may be conveyed thither in the torrent of the circulation, from some other part in a state of suppuration.

*Lesions of the blood in the cavities of the heart.* It is now generally admitted by pathologists, that some of the coagula found after death in the heart, have been formed there during the life of the individual. The great consistence of these coagula, their close adhesion to the substance of the heart, and sometimes even their evident organization, fully establish the fact that blood may, during life, coagulate in the cavities of the heart, and there become the nidus of several morbid alterations.

"The intimate connexion that is formed between the coagulum and the heart is the simple consequence of a very general law, by virtue of which, two parts endowed with life cannot remain in contact without being united to each other, by a process somewhat resembling that by which grafting takes place in the vegetable kingdom.

"The formation of vessels in the coagulum is the result of another law in the animal economy, namely, that every particle of blood which remains in a state of stagnation in the living body has a constant tendency to become organized." (P. 351.)

If once we admit that polypous concretions in the heart may become organized, it follows, as a necessary consequence, that they may have different morbid productions formed in their interior. M. Andral relates several cases in which pus was found in these concretions; and though it is admitted that, in some, the pus was not actually formed in the coagulum, but that it was brought into the heart with the blood in which it was circulating, yet there are others in which the pus must have been formed in the coagulum.* Cartilaginous and osseous productions have been known to be developed in the interior of these polypous concretions. M. Andral cites several cases of this kind. The fact of the organization of polypi of the heart affords, likewise, the most probable explanation of the formation of those vegetations, which are occasionally found on the inner surface of the heart, and on the valves, and which assume various appearances. "Thus, some bear a striking resemblance to venereal crusts, others to those syphilitic productions named cauliflower excrescences, others are like strawberries, and others assume a cylindrical or tapering form." The causes which influence the formation of vegetations in the heart, are not always easily appreciated: in general, however, they may be found either in the blood itself, or in the heart.

"The blood may become so modified as to have a constant tendency to coagulate in the interior of the heart, whenever the circu-

* "I have seen a preparation," says Mr. Wardrop, (Works of Dr. Baillie, vol. ii. p. 20, note,) "of a polypus in the left ventricle, in the centre of which was a distinct abscess."

lation experiences the least impediment, or even independently of any such impediment: this will appear less surprising, when we recollect that the heart does not wholly discharge its cavities at each contraction, but that a certain proportion of blood remains behind, and is (as it were) filtered through the meshes of the net-work which lines the internal surface of each of the heart's cavities. Certain morbid conditions of the heart may likewise favor the formation of these coagula, by impeding the free passage of the blood through its cavities. Thus, any alteration in the thickness of the parietes, the dimensions of the cavities, or the structure of the orifices, must necessarily produce a greater or less degree of stagnation of blood in the heart, and consequently afford a greater facility for its coagulation. In like manner, if the internal membrane be irritated, and, in consequence of that irritation, throws out a coating of lymph, which renders its surface rugged and uneven, the blood, in passing over the uneven surface, will have a constant tendency to form depositions on it, as it does in the arteries, when their internal membrane has lost its natural polish. The explanation of this fact on mechanical principles is, perhaps, not altogether satisfactory; no doubt, however, can be entertained of the fact, that wherever the lining membrane of the sanguiferous system has been irritated and lost its natural polish, it sooner or later becomes coated with a layer of coagulated blood. Whether the irritated part acts on the blood mechanically or vitally, is, in the present state of our knowledge, difficult to determine; in the latter supposition, there is, however, nothing more surprising, than in that singular modification of the blood, termed its buffy coat, which we so constantly observe in cases of pleuritis and inflammation of the joints." (P. 358.)

*Lesions of the innervation of the heart* admit but of very brief discussion in a work dedicated to pathological anatomy, however fertile the subject might be for consideration in a purely practical point of view. Diseases of this class do not depend on any alteration of structure cognizable to our senses. The functions of the heart may be so deranged, without any structural injury, as to give rise to many of the same symptoms, and sometimes even to the fatal consequences, which result from structural alteration. The following extract will afford the inexperienced practitioner some useful hints. If he weighs, with due attention, the statements of M. Andral, which we take upon ourselves to affirm are strictly correct, he will be little likely to fall into practical errors, which are but too common and too mischievous.

"It will readily be admitted that a simple modification of the nervous influence, independently of any organic alteration, may so derange the heart's action as to cause palpitations; for who has not felt his heart palpitate under a strong mental excitement?

These nervous palpitations occur principally in three general conditions of the economy, which it is important to distinguish carefully, as each requires a different plan of treatment.

"The first of these conditions is a state of plethora, which causes the heart to beat with too much force, just as it produces vertigo, dizziness, &c. Low diet and copious venesection are here indicated.

"The second of these conditions is a state of anæmia. Palpitations, accompanied by more or less of dyspnœa, are a frequent source of uneasiness to invalids who have been submitted to too severe a regimen, or kept too long on low diet: in all such cases, these symptoms are invariably relieved by the use of a more generous diet, which, we may suppose, acts by replenishing the blood, and invigorating thc system. An analogous case to the preceding is that of animals falling into convulsions from excessive hemorrhage. But there are likewise certain individuals who have neither been kept on too low diet, nor bled to excess, but who habitually and constitutionally make less blood than others, and are constantly subject to palpitations and dyspnœa connected with the anæmic state of their system. In such cases, shall we attempt to remove the palpitations by bloodletting, on the hypothesis of relieving a local plethora of the heart, of the existence of which we have no proof whatever; or shall we not rather fulfil the evident indication of restoring the balance of the system by endeavouring to increase the absolute quantity of the blood, or the relative proportion of its fibrine and colouring matter? Certain it is, that under the treatment calculated to fulfil the latter indication, we see those palpitations disappear, which would infallibly have been aggravated by venesection and low diet.

"Lastly, the third condition of the economy, which we have said is often accompanied by palpitations, does not depend either on an excess or deficiency of blood, for that fluid appears to exist in its due proportion, but on some primitive alteration in the action of the nervous centres themselves. In some cases of this kind, the palpitations are the only symptoms of the morbid state of the nervous system; in others, they are only one of the derangements in which almost every organ in the body more or less participates. Let us take, for example, a young hysterical girl, in whom the most extraordinary derangements in all the functions succeed each other with the greatest rapidity. If we bleed her, we neither diminish the palpitations nor alleviate the other symptoms of her complaint. If we give her tonics, our success is no better. What then remains for us to do, but to employ such means, whether medical or moral, as act neither by debilitating nor yet by strengthening the system, but by substituting a new modification of the nervous system in the place of the old one. In this way, I conceive, may be explained the efficacy of matrimony in at once removing a whole train of morbid phenomena, which had been combated in vain by different articles from the Materia Medica. I do not so much allude to the physical effects of marriage, as to the moral influence it exerts on the mind of the young woman, by the various emotions it excites, and by the complete revolution it works in all her ideas, habits, and pursuits." (P. 362.)

We now arrive at the description of "*Diseases of the Arteries.*" Various arguments and experiments are adduced to shew that the internal surface of the arteries may be of a bright red colour, and still that no inflammatory action may have existed during the life of the individual. "If we wash an artery, slit it open, and expose its internal surface to the air, its inner membrane soon turns red, and the subjacent coats acquire a bright vermilion colour." Upon this subject, M. Andral comes to the conclusion of most modern pathologists,* that but little value can be attached to the red colour, as a mark of arterial disease, unless where it is accompanied by some other morbid alteration. From various lesions of nutrition in the coats of the arteries, these vessels may have their dimensions greatly altered: their caliber may be either increased, diminished, or totally obliterated. Each of these alterations is considered separately. An artery may be dilated in its entire circumference, or in only a part of it.

"The dilatation of an artery in a part only of its circumference, is so rare an occurrence that some authors have questioned its reality: the fact, however, is placed beyond dispute, for on more occasions than one I have been able to trace distinctly the three arterial coats passing over the walls of a sac which seemed as if appended to the artery with the cavity of which it communicated." (P. 376.)

This dilatation of an artery, without rupture of any of its coats, has received from authors, says M. Andral, the name of *true aneurism.* We are somewhat surprised that no reference is made to the disputes that have arisen upon this pathological point. SCARPA, for instance, revives the old doctrine, and makes a distinction between dilatation of an artery and aneurism. He believes that aneurism never consists in dilatation, but that it always arises from rupture of the proper coats of an artery, and the injection of arterial blood into the investing sheath of the vessel. For a full discussion of this subject, we refer to Mr. HODGSON's work on the Arteries.

The pathology of aneurismal tumors is very briefly entered into by M. Andral. A narrowing of the arteries may exist, either as a congenital alteration, or as the effect of disease. The obliteration of an artery may be effected in various ways:

* Vide CRAIGIE's Elements of General and Pathological Anatomy, p. 89; or our review of the work, in the number for March 1829, p. 251.

"Thus, in some cases we find only a ligamentous cord, such as is formed in the adult by the umbilical artery; in others, the point where the artery is obliterated is occupied by coagula of fibrine, more or less organized, adhering firmly to the parietes of the vessel, and sometimes incorporated with them; whilst in other cases, again, the obliteration results from the complete obstruction of the cavity of the artery by osseous concretions." (P. 385.)

The first species of obliteration has been twice observed in the aorta. In both cases there had been present all the symptoms of organic disease of the heart, and in both the circulation was kept up by the aid of collateral arteries, considerably dilated. The second kind of obliteration has been repeatedly found in the arteries of the lower extremities, in cases of *gangræna senilis,* and it is reasonable to conclude that, in such instances, the obliteration of the arteries is the cause of the gangrene.

Upon the subject of ossification of the arteries, M. Andral offers some ingenious, and, as far as we know, novel speculations.

The next section treats of "*Diseases of the Veins.*" It is laid down as a general principle that, in the veins, still more than in the arteries, the existence of redness, unaccompanied by other morbid changes, cannot be considered as a proof of disease. A general and interesting sketch is given of all that is yet known of the various lesions to which the veins are subject.

We must pass over the next chapters, which describe the different diseases of the *spleen* and *lymphatic system,* with the notice of one brief remark. A great proportion of the melanic tumors described by writers in the human subject, and in animals, appear to the author to be nothing else than lymphatic ganglions coloured black.

"*Respiratory Apparatus.*" Under this head is included a minute description of the various morbid appearances which are found in the air tubes, and the parenchyma of the lungs. While we admit the great importance of every part of these subjects, and the very able manner in which they are discussed by M. Andral, we are compelled to confine ourselves to those remarks which appear to us of the most practical importance.

The mucous membrane of the air-passages presents two species of thickening: the first depends chiefly on the membrane being in a state of congestion; the second is produced by a preternatural degree of activity in the nutrition of the membranous tissue. The first species of thickening may occur at any point of the mucous membrane, but is most constantly observed in the larynx and in the small bronchial tubes.

"The tumefaction of that part of the mucous membrane which lines the margin of the glottis is sometimes so considerable, especially in children, in whom the aperture is particularly small, as to block it up almost completely, and consequently to oppose the free passage of the air into the lungs, and so produce all the symptoms of croup except the membraniform expectoration. I am disposed to think, that this form of croup is, at the least, as common as that which depends on the formation of false membranes; and it certainly accounts more satisfactorily for the peculiar croupy symptoms, the great dyspnœa, the peculiar ringing sound of the voice and cough, and the no less peculiar sound which the column of air makes in its passage through the larynx." (P. 470.)

The mucous membrane of the air-passages, like that of the intestines, may become preternaturally softened; and the author has been more than once surprised to find no other lesion in the larynx than this softening of its mucous membrane, in individuals whose voice had been for a length of time either hoarse or altogether extinct. Ulcers of the larynx are more common than those of the trachea or bronchia; "but, as it seldom happens that we find ulcers in the larynx, on dissection, without at the same time finding them also in the parenchyma of the lungs, we can scarcely form a correct idea of their effect on the general health." The air-passages are sometimes, more frequently indeed than any other mucous cavity, lined with membraniform concretions. Much importance has been deservedly attached to the local irritation which precedes or accompanies the developement of these false membranes; but we are properly urged never to forget that a principal cause of their formation is to be found in the state of the general health, and consequently that, in treating these affections, copious bloodletting is not in every case the only indication to be fulfilled. The abstraction of blood is, within certain limits, of infinite utility in subduing the local affection; but, when carried to excess, it may favor that state of the system, of which the local affection is often merely the effect.

"It is certain that the formation of false membranes may be readily produced by introducing into the air passages any highly irritating substance, such as alcohol, dilute sulphuric acid, oil of turpentine, &c.; they have likewise been produced by inspiring chlorine or ammonia for any length of time; but these results do not constantly follow; the action of these irritants on the mucous membrane of the trachea or larynx does not in every case determine the formation of a false membrane; consequently, there must be a predisposition on the part of the individual. But, if this predisposition be very strong, it is evident, that the action of much less irritating substances than any of those above mentioned, nay, the slightest degree of irritation, will be sufficient to determine the formation of false membranes in the larynx, trachea, and bronchia: whereas, if there be no predisposition to the disease, the highest degree of artificial irritation that we can excite, or the most intense inflammation developed of its own accord, will not be sufficient to cause the formation of a single false membrane.

"Hence I think it is evident that the formation of false membranes in the air passages cannot be accounted for solely by the intensity of the irritation which preceded their developement. Is it because children are subject to more violent irritation of the air passages than adults, that the formation of false membranes is so much more common at that period of life? Certainly not; but rather because there is in children a peculiar state or disposition of the constitution, which causes any irritation that occurs to present a certain train of symptoms, follow a certain course, and terminate in a certain manner. Is it because two blisters create different degrees of irritation, that one causes a secretion of pus, while the other produces a thick layer of a substance like hog's lard all over the blistered surface? Such an opinion is merely hypothetical; whereas all practitioners are well aware that the difference of the secretion coincides more frequently with certain general conditions of the economy, which experience teaches us to recognize, than with any determinate degree of irritation in the blistered surface. In some children the cause which influences the formation of these false membranes in the air passages is evidently general or constitutional, inasmuch as they are often formed at the same time in the nasal fossæ, in the alimentary canal, around the anus, in the external meatus auditorius, and wherever the skin has undergone the slightest solution of continuity." (P. 483.)

Calculi are sometimes found in the bronchia: the cause of their formation, M. Andral says, is no better understood than that which produces their developement in other parts of the body; "but this much, at least, is certain, that their production cannot be accounted for by irritation."

After having given a very minute detail of the morbid anatomy of the respiratory organs, M. Andral proceeds to describe the diseases of the secretory apparatuses, lesions of the secreting tissue, lesions of the fluid secreted by the serous and cellular membranes, and diseases of the glandular organs of secretion.

In the latter section, the *diseases of the liver and its appendages* are first considered, and next *diseases of the urinary apparatus.* It has been supposed, by some pathologists, that abscesses of the liver may arise in consequence of irritation, not of the liver itself, but of the brain. M. Andral, however, thinks that this fact requires further investigation. To another class of abscesses of the liver

"Belong those cases in which the pus seems to have been carried into the liver in the blood by a genuine metastasis. In these cases, at the same time that we find one or more purulent collections in the hepatic parenchyma, without any appreciable alteration of the surrounding tissue, we meet with similar collections in other parenchymatous organs. This may occur in three different cases: 1, where there exists a collection of pus in some other part of the body; 2, immediately after such a collection has been dried up; and, 3, after the establishment of a suppuration in some part of the body by a surgical operation. In these three cases, we may account for the phenomenon, either by admitting a metastasis of the morbid product; or by supposing that, from the circumstance of pus being no longer formed in a part of the system where it had long been secreted, the system, being accustomed to the secretion, set it up in another part; or by supposing that there are certain individuals in whom suppuration cannot be established in any one point of their body, without having a tendency to become established in other points also." (P. 599.)

We cannot establish any connexion between the alterations of the liver (such, at least, as we can discover by anatomy,) and those of the bile. In the greater number of the diseases of the liver described, the bile in the ducts and gall-bladder does not appear altered in quantity or quality. The structure of the liver may not be at all changed, and still the bile may be excessive or deficient in quantity, or altered in its sensible qualities.

"The reason of the secreting fluids being altered without the secreting organs being so, is, that, in the liver, as in every other organ destined to separate a fluid from the blood, the alterations of texture that are apparently the most serious, are not always those that exert the greatest influence over the act of secretion; the derangement of this secretion seems to depend rather on certain lesions of the organ that escape our observation, and not unfrequently on the lesions of other parts. Thus, Magendie's experiments have proved that, by changing the food of an animal, we can alter at pleasure the composition of the bile, which is evidently owing to the previous alteration of the blood from the same cause." (P. 607.)

Practitioners are too apt to infer, when a patient is jaundiced, that there must necessarily be some hepatic disease. The following remarks shew the fallacy of this opinion:

"It was supposed that the jaundice always arose from some obstacle in the biliary ducts to the passage of the bile into the duodenum; but this opinion is incorrect, inasmuch as those ducts are often found perfectly free in persons that die of the disease. Indeed nothing can be more variable than the state of the liver in jaundice; any one of the numerous alterations to which that organ is subject, may be attended by it, but none of them are constantly or inseparably connected with it. There are even cases of icterus where we cannot discover any lesion whatever in the liver or its appendages; and in many such cases we have reason to doubt that the liver had any thing to do with the disease. We are not to suppose, however, that the yellow tinge of the skin can be produced only by the presence of the colouring matter of the bile in the blood, as it sometimes seems to arise merely from a sanguineous suffusion of its tissue. Such, especially, seems to be the nature of the icterus neonatorum, in which we can observe the red tinge of the skin gradually changing into a yellow, which in its turn fades away, and is succeeded by the natural colour of the part. Neither can we find in the liver of children that die of this disease any constant lesion that could account for it. Some have asserted, indeed, that in such cases they found the liver gorged with blood; but it is found at least as frequently in the same state where there has been no jaundice at all." (P. 610.)

There is a curious and well-known fact, which bears immediately upon this subject, to which M. Andral has not adverted. Jaundice has been suddenly produced by powerful mental emotions: an example of this kind fell under our own notice. Is it probable that the liver is affected in such instances? we think not.

M. Andral prefaces his observations upon the *diseases of the urinary apparatus,* by remarking that, although it is influenced by a great variety of disturbing causes, its organization is not very frequently altered. In the greater number of its diseases, whether acute or chronic, we cannot discover, by dissection, any change in the structure of the kidneys, and the rest of the apparatus is equally free from lesion; so that we have here another case, in addition to others which have been before mentioned in the progress of the work, where the functional derangements of an organ are not elucidated by any obvious structural alterations. The following observation is important in reference to the pathology of diabetes.

"It has been often asserted that the chief alteration observed in the kidneys of persons that die of diabetes, is an extreme paleness of their tissue. However, I have just mentioned a case in which they were, on the contrary, in a state of hyperæmia. In another case that fell under my observation, they retained their natural appearance. We shall see further on, some cases in which the disease was accompanied by hypertrophy of the kidneys. As far as I am aware, there has not been a single instance recorded, within the last ten years, of the kidneys of a diabetic person being in that state of anæmia that has been so insisted upon by some authors." (P. 614.)

Morbid granulations have been described in the cortical substance of the kidney. M. Andral bears his testimony to the excellent description which Dr. Bright has given of this species of disease.

"In the various cases of this affection observed by Dr. Bright, the urine contained albumen, and the patient was dropsical, although the heart and liver were sound. I have mentioned a similar case in the third volume of my Clinique Médicale, which appeared about a year before the publication of Dr. Bright's work. It is difficult to comprehend how this state of the kidneys should produce dropsy, but the fact is certain." (P. 617.)

But a short space is devoted to the *diseases of the generative apparatus*, as a great many of them belong to the pathology of the exterior, and the plan of the work allows the author to dwell only upon those lesions which are more particularly connected with the pathology of the interior.

*Diseases of the breast*, and *diseases of the fœtus and its appendages*, are next described.

The work concludes with an account of the various lesions to which the *nervous system* is obnoxious. We regret that our limits prevent us from giving more than the following introductory observations, which contain another proof that M. Andral is not led, by over enthusiasm for that department of medical science to which he has so richly contributed, to the erroneous conclusion which some zealots in the cause of morbid anatomy have arrived at, namely, that no disease can exist in the living, the signs of which cannot be detected by the scalpel in the dead.

"If the variety of the functional derangements of an organ bore any constant proportion to that of its derangments of texture, no part should present a greater variety of lesions than the nervous centres and the nerves: such, however, is not the fact; these lesions are few in number, and frequently bear no proportion to the nature or intensity of the symptoms; nay, it sometimes happens that we cannot discover any lesion whatever in the nervous system, although its functions have been seriously deranged during life. It is, however, highly probable that some organic lesions do exist in such cases, though they escape our notice; and as there are few functional disorders of the brain or other parts of the nervous system which may not thus occur without any appreciable lesion, it follows that, when we do find an alteration of structure in those parts, we ought to be cautious how we attribute the functional derangement to it, inasmuch as such alteration is often purely accidental, secondary, or consecutive, and the derangement of function depends on some other lesion which altogether escapes our notice. What strengthens this view of the subject is, that we find that a lesion which produces certain symptoms in one case, does not produce any symptom at all in another; and that the same lesion may be accompanied by the most dissimilar symptoms, and the most dissimilar lesions by the same symptoms.

"Accordingly, notwithstanding the ingenious researches which have lately been made in this department of pathology, we must for the present observe considerable caution and reserve in our attempts to explain the derangements of the functions of the nervous system observed during life, by the nature of the organic lesion found after death. Neither can we come to any more positive conclusion from the situation of the lesion; for, morbid anatomy has but rarely confirmed the conclusions relative to the functions of the various parts of the nervous system drawn from experimental physiology, or comparative anatomy; while, on the contrary, it has often invalidated them." (P. 709.)

We know no work on pathological anatomy that will be studied with greater advantage by the profession than that of which we have now completed the analysis. M. Andral makes no vain attempt to shew that the morbid anatomist can *always* detect the nature of disease by his *post-mortem* examinations. The facts that have yet been developed by pathological anatomy are clearly and accurately stated, and where this valuable auxiliary to medical science still leaves us in doubt, its imperfections are confessed with the most laudable candour.

---

*Change of Air, or the Pursuit of Health; an Autumnal Excursion through France, Switzerland, and Italy, in the Year* 1829; *with Observations and Reflections on the Moral, Physical, and Medicinal Influence of Travelling-Exercise, Change of Scene, Foreign Skies, and Voluntary Expatriation.* By James Johnson, M.D. Physician Extraordinary to the King.—8vo. pp. 294. London: S. Highley, and T. and G. Underwood, 1831.

If every man, who flies to foreign climes for recreation of mind and body, after having lived beyond his income of health in this "overgrown wen of a metropolis," either from anxieties of business, or the perhaps more exhausting avocation called "pleasure," were capable of giving to the world as amusing and instructive a volume as that before us, we might, perhaps, wish that the number of those who change the air in pursuit of health was much greater than it is: but, unfortunately, too many travellers either tell us nothing but what has been told before, or dwell minutely upon facts which, as they are incidental to all people and all countries, might easily have been imagined, if they had never been told at all. Too often are their readers conducted "through wet and dry, over rough and smooth, without incident and without reflection." Not so with Dr. Johnson: although his course was rapid, he has collected much interesting and amusing information, and, in his descriptions of the different countries he passed through, and the many objects of curiosity which attracted his notice, he has preserved a freshness and originality which reflect high credit upon his talent as a writer, and as an acute observer, considering the many pens that have before worked up the same materials. The volume also contains much useful medical information, which the travelling invalid, who seeks for instruction on his own account, as well as the practitioner, who may be called upon to impart it to others, will read with much advantage. Dr. Johnson has evidently, upon this occasion, written in that light and cheerful temper of mind which seeks to combine amusement and instruction: the reader will not, therefore, expect to find that tone of gravity which well becomes a professed medical treatise, but which would be out of place in "Change of Air for the Pursuit of Health."

The work consists of three parts.

"The *first* contains some observations on that *wear* and *tear* of mind and body, which we particularly remark in civilized life, and especially in large cities; together with some suggestions as to the antidote or remedy. The *second* part consists of reflections and observations made during an excursion through France, Switzerland and Italy, in the year 1829; partly for recreation, but principally for renovation of health. The *third* division contains some remarks and speculations on the moral, physical, and medicinal influence of foreign, and especially of an Italian climate and residence, in sickness and in health. In each of these divisions, the author hopes that he has been able to combine utility with some portion of amusement, for those (and they are many) who, like himself, seek an occasional renovation of health, in a temporary relaxation from the toils and cares of avocation." (*Pref.*)

We must confine ourselves to the notice of those subjects which come fairly within the limits of a medical journal; but, in thus limiting our account of the work, we must, in justice to the author, confess that we pass over altogether many very interesting chapters, which contain abundant materials for a detailed article in periodical publications devoted to general literature.

At the commencement of the volume, a few observations are made upon that "wear and tear" of the living machine, mental and corporeal, which results from overworking the intellectual faculties, rather than the corporeal powers. Anxiety of mind and bad air powerfully predispose to this condition of body and mind, which Dr. Johnson considers to be "intermediate between sickness and health." We know well what this "wear and tear" is, and we are inclined to consider it as very nearly allied to positive disease. If it be between sickness and health, it is certainly much nearer the former than the latter. Dr. Johnson says, this "wear and tear" complaint is almost peculiar to England, and that it is probably a descendant of the old "English malady," about which so much was written a century ago. "And why should it predominate in London so much more than in Paris? The reason is obvious: in London, business is almost the only pleasure; in Paris, pleasure is almost the only business." That "the wear and tear of avocation induces the semblance, if not the reality of premature old age," will not be denied by any one who looks around him upon those who work their minds incessantly without giving up some portion of their time to active bodily exercise. If we except a few of the public schools, in which, we know, much too laborious duties are imposed upon the youthful mind, we are not inclined to believe, with Dr. Johnson, that, in the systems of male and female education, there is either "terrible competition" or injurious "struggles for pre-eminence." To a certain extent, both the one and the other are very desirable, and we think, in general, they are not pushed beyond the bounds of judgment and discretion. We agree with him, that "the march of intellect is sometimes a *forced* march;" and that, when it is so, it must be destructive to the health and happiness of those who are imprudently urged beyond the rational exercise of their mental faculties.

After having glanced at some of the more prominent features of the "wear and tear" of civilized, and especially of civic life, the author proceeds to point out the antidote. An autumnal excursion is advised, and "it is probably of little importance to what point of the compass the tourist steers his course." To shew the salutary effects of travelling, a long extract is given from a well-known work, recently published, by Dr. Johnson, "on Indigestion."

The author now enters "the Steamer," and starts upon his peregrination. We would willingly accompany him step by step, but the purposes of our Journal will not admit of our following him in his very agreeably described excursion. Suffice it to say, that, in his course through France and Switzerland, he gathered much interesting information, both as to the characteristics of the people and their countries.

We take him up at "Sion;" and here the important subject of *cretinism* is discussed. We shall give rather a long extract from this part, as a specimen of the general style of the volume.

"We are now in the centre of the Vallais, the head-quarters of goitre and cretinism. There are few portions of the earth's surface, in these temperate climes, better calculated for the deterioration, if not the destruction, of life, than the Valley of the Rhone. It is bounded on each side by steep mountains, four or five thousand feet in height, and the intermediate ground contains all the elements that are found to operate against human health. The valley consists, in some places, of a rich, flat, alluvial earth, covered with corn, fruit trees, and gardens; in others, it presents swamps and meadows; then, again, jungle and woods, vineyards, pine forests, &c., while brawling brooks intersect it in all directions, and often inundate it, in their precipitous course from the mountains to the Rhone, which runs through its centre. Were this valley beneath a tropical sun, it would be the seat of pestilence and death. As it is, the air must necessarily be bad; for the high ridges of mountains, which rise like walls on the north and south sides, prevent a free ventilation, while, in summer, a powerful sun beats down into the valley, rendering it a complete focus of heat, and extricating from vegetation and humidity a prodigious quantity of malaria. In winter, the high southern ridge shuts out the rays of a feeble sun, except for a few hours in the middle of the day, so that the atmosphere is not sufficiently agitated at any season of the year. To this must be added, the badness of the waters, which, along the banks of the upper Rhone, are superlatively disgusting.

" As the Vallais is the land of cretinism, so is Sion the capital of that humiliating picture of humanity! There are but few travellers who take the trouble to examine Sion philosophically, and make themselves acquainted with the state of its wretched inhabitants. I explored this town with great attention, traversing its streets in every direction; and I can safely aver that, in no part of the world, not even excepting the Jews' quarter in Rome, or the polluted back lanes of Itri and Fondi, in the kingdom of Naples, have I seen such intense filth? With the exception of two or three streets, the others present nothing on their surface but a nameless mass of vegeto-animal corruption, which, in all well-regulated towns, is consigned to pits, or carried away by scavengers. The alleys are narrow; and the houses are constructed as if they were designed for the dungeons of malefactors, rather than the abodes of men at liberty.

" Goitre, on such a scale as we see it in the Vallais, is bad enough; but cretinism is a cure for the pride of man, and may here be studied by the philosopher and physician on a large scale, and in its most frightful colours. This dreadful deformity of body and mind is not confined to the Alps. It is seen among the Pyrennees, the valleys of the Tyrol, and the mountains of China and Tartary. Nearly 200 years have elapsed since it was noticed by Plater, in the spot where I am now viewing it; but Saussure was the first who accurately described this terrible degeneracy of the human species. From common bronchocele, and a state of body and mind bordering on health, down to a complete destitution of intelligence and sensibility, in short, to an existence purely vegetative, cretins present an infinite variety of intermediate grades, filling up these wide extremes. In general, but not invariably, goitre is an attendant on cretinism. The stature is seldom more than from four to five feet, often much less; the head is deformed in shape, and too large in proportion to the body; the skin is yellow, cadaverous, or of a mahogany colour; wrinkled, sometimes of an unearthly pallor, with unsightly eruptions; the flesh is soft and flabby, the tongue is large, and often hanging out of the mouth, the eyelids thick; the eyes red, prominent, watery, and frequently squinting; the countenance void of all expression, except that of idiotism or lasciviousness; the nose flat; the mouth large, gaping, slavering, the lower jaw elongated; the belly pendulous; the limbs crooked, short, and so distorted as to prevent any thing but a waddling progression; the external senses often imperfect, and the cretin deaf and dumb, the *tout ensemble* of this hideous abortion of Nature presenting the traits of premature old age? Such is the disgusting physical exterior of the apparently wretched, but perhaps comparatively happy, cretin!

"If we look to the moral man, (if man he can be called,) the picture is more humiliating; the intellectual functions being, as it were, nul, certain of the lower animal functions are in a state of increased activity. The cretins are voracious and addicted to low propensities, which cannot be named. To eat and to sleep form their chief pleasures. Hence we see them, between meals, basking in nonchalance on the sunny sides of the houses, insensible to every stimulus that agitates their more intelligent fellow-creatures, frequently insensible to every call of Nature itself!" (P. 55.)

Passing on from this melancholy example of the effects of climate, or, at all events, of physical agencies, on the moral and corporeal constitution of man, the author next inquires into the causes which are supposed to produce them.

"In the first place, it is remarked that cretinism is bounded to certain altitudes above the level of the sea. The Vallais itself, and the ravines or gorges of the mountains by which it is enclosed, are the chief seats of this deformity. All, or almost all, those who inhabit the higher ranges of the mountains overlooking the valleys are exempt from the malady. This single fact proves that cretinism is owing to a physical rather than a moral cause, or series of causes. There can be no material difference in the moral habits of peasants residing at the base and on the brow of the same mountain. If the former be more subject to goitre and cretinism than the latter, it must be owing to something in the air they breathe, the water they drink, or the emanations from the soil on which they reside. Saussure, Ferrus, Georget, and all those who have personal knowledge of the subject, acknowledge that, at a certain height (five or six hundred toises) among the Alps, goitre and cretinism disappear. In the year 1813, M. Rambuteau, then prefect of the department of the Simplon, addressed a memoir to the minister of the interior of France, on this subject, in which, after describing very accurately the medical topography of the Vallais, with its malarious exhalations, stagnant atmosphere, and alternate exposure to the rays of a burning sun, and piercing icy winds, as the causes of cretinism, goes on to add, 'the use of waters, which, in descending from the mountains by long and circuitous routes, become impregnated with calcareous salts.' 'A ces causes il croit devoir adjuter l'usage des eaux, qui, en descendant des montagnes et parcourant de longues distances, se chargent de sels calcaires.'* As moral auxiliaries, the prefect enumerates 'the indolence of the inhabitants, their want of education, the dirtiness of the houses, the badness of the provisions, their drunkenness and debauchery.' M. Rambuteau mentions some curious particulars respecting this dreadful deterioration of human nature. He affirms that those Valaisans who intermarry with the Savoyards from the Italian side of the Alps, give birth to more cretins than those who form matrimonial connexions with the inhabitants of their native valley. The females of the latter place, who marry men born on the higher regions of the Alps, and who are accustomed to live in the open air, with much bodily exercise, hardly ever bring forth cretinous children. The same intelligent observer remarks that, 'Wherever cretinism is seen, goitre is also prevalent, but the latter is found in places where the former does not exist.' Hence he is led to the conclusion, that 'the nature of the two maladies is the same, (le principe des deux maladies est le même,) but the cause is more active where cretinism and goitre both prevail, more feeble where goitre only obtains.' In short, we find in the Vallais, and in the lower gorges or ravines that open on its sides, both cretinism and bronchocele in the most intense degrees; as we ascend the neighbouring mountains, cretinism diappears, and goitre only is observed, and when we get to a certain altitude both maladies vanish, and the Alpine peasant or shepherd once more assumes the 'image of his Creator!'

"It is said and believed by travellers, that cretinism is decreasing in the Vallais. The diminution is, I fear, more apparent than real. The 'march of intellect,' and the intercourse with strangers, have taught the parents and friends of these wretched creatures to doubt that the cretin is the favorite of Heaven, as is thought of idiots in Turkey. They, therefore conceal, rather than expose, their offspring so afflicted. I saw them driving them in from the back streets of Sion on my approach. It is probable, however, that

* "Dr. Bally, a native of a goitrous district in Switzerland, states the following very important fact. 'Bronchocele appears to me to be produced by certain waters which issue from the hollows of rocks, trickle along the cliffs of mountains, or spring from the bowels of the earth. That this is the case, I may instance some fountains in my own country, (Department du Leman, au Hameau de Thuet,) the use of whose waters will, in eight or ten days, produce or augment goitrous swellings. Such of the inhabitants of the above village as avoid those waters are free from goitre and cretinism.'" (Dict. des Sciences Medicales, t. vii.)

there is a diminution in the number of cretins in the Vallais. Many of the auxiliary causes are on the decline. The people are becoming more sober, more industrious, more cleanly. Those who can afford the expense, also, send their children up into the mountains to check the tendency to cretinism.

"Enough has been said, and a great deal more will be shewn hereafter, to prove the influence of climate and locality on the corporeal and intellectual constitution of man: and I hope to convince John Bull, in the course of our wanderings together, on this little tour, that all the moral and physical evils of the world are not included in fogs and taxes, against which he so bitterly complains in his own country." (P. 57.)

With respect to the causes of goitre, there are almost as many opinions as there are cantons in Switzerland; such as rapid atmospherical changes, intense heat in some valleys, intense cold in others; stagnant air, also, especially about Martigni, from the position of the mountains; and, lastly, some peculiar quality of the water used by the inhabitants. We may just mention one or two facts which go to prove that the disease may, with much probability, be attributed to some noxious principles in the water. About ten years ago, a friend of ours anxiously sought to obtain information upon the subject of goitre. He was informed by the inhabitants of Romont, a small town in a hilly but not mountainous country in the north of Switzerland, that they remembered a period when that malady did not exist there: but, in consequence of the town becoming more and more enlarged, the governor's well, from whence the people were always supplied with water, became inconvenient, from its locality and distance: pipes were consequently laid down from a more conveniently situated and nearer spring; and in a short period from the time when the people were supplied with water from this source, goitre appeared among them.

We must pass over that "seventh wonder" of the world, the Simplon road, an accurate survey of which did not disappoint Dr. Johnson, although "he had strong presentiments that it would do so," and take him up at the great hospital of Milan, where we, of course, find medical matter. *Pellagra* is the subject of the author's comments.

"This horrible malady, or complication of maladies, has only been observed during the last sixty or eighty years, and is rapidly increasing. The proportion of cases in the hospital is very considerable. It begins by an erysipelatous eruption on the skin, which breaks out in the spring, continues till the autumn, and disappears in the winter, chiefly affecting those parts of the surface which are habitually exposed to the sun or the air. This cutaneous symbol of an internal disorder is accompanied or preceded by remarkable debility, lassitude, melancholy, moroseness, hypochondriacism, and not seldom a strong propensity to suicide. Year rolls on after year, and the cutaneous eruption, as well as the general disorders, become more and more aggravated, with shorter and shorter intervals in the winter. At length the surface ceases to clear itself, and becomes permanently enveloped in a thick, livid, leprous crust, somewhat resembling the dried and black skin of a fish! By this time, the vital powers are reduced to a very low ebb, and not seldom the intellectual functions. The miserable victim of this dreadful pellagra loses the use of his limbs, more particularly of the inferior extremities, is tormented with violent colic, headach, nausea, flatulence and heartburn, the appetite being sometimes nul, at others voracious. The countenance becomes sombre and melancholy, or totally void of expression, the breath fetid, the teeth rotten, the inside of the mouth ulcerated, the mucous membrane highly irritable, and diarrhœa is a common accompaniment of the other disastrous train of miseries. But the most distressing phenomenon of all, is a sense of burning heat in the head and along the spine, from whence it radiates to various other parts of the body, but more especially to the palms of the hands and soles of the feet, tormenting the wretched victim day and night, and depriving him completely of sleep! He frequently feels as if an electric spark darted from the brain, and flew to the eyeballs, the ears, and the nostrils, burning and consuming those parts. To these severe afflictions of the body are often added strange hallucinations of the mind. The victim of pellagra fancies that he hears the incessant noise of millstones grinding near him, of hammers resounding on anvils, of bells ringing, or the discordant cries of various animals! The disease, when advanced, takes the form of many other maladies, as tetanus, convulsions, epilepsy, dropsy, mania, and marasmus, the patient ceasing at last to exist and to suffer, when reduced to the state and appearance of a mummy. It is by no means uncommon,—who can say it is wonderful,—that the wretched being abbreviates the term of his afflictions, and anticipates the too-tardy hand of death in a paroxysm of suicidal mania! It is remarkable that this tendency to self-destruction very often assumes the form of a desire to consummate that last act of the tragedy by drowning, so much so, that Strambi, a writer on the pellagra, has given it the name of hydromania, when this propensity exists.

"Whatever may be the precise nature of the cause of this dreadful disease, it is certain that it is almost universally confined to to those who reside in the country, leading an agricultural life, and to the lowest orders of society. It is not bounded by any age, being frequently seen in the youngest children. The whole of the flat country on both sides of the river Po, but more especially the fertile and level plains between that river and the Alps, are the theatre and head-quarters of pellagra. I have only sketched the more prominent features of the complaint, and I have by no means magnified either its horrors or its prevalency. If those who doubt this statement will consult the native writers on the malady, as Strambi, Trapolli, Soler, Zanetti, and many others, they will acknowledge that I have softened rather than exaggerated the picture." (P. 75.)

"The cause of this frightful endemic has engaged the pens of many learned doctors. But it is just as inscrutable as the causes of hepatitis on the coast of Coromandel, elephantiasis in Malabar, Beriberi in Ceylon, Barbadoes leg in the Antilles, goitre among the Alps, the plica in Poland, cretinism in the Vallais, or malaria in the Campagna di Roma! It is an emanation from the soil; but whether conveyed in the air we breathe, the food we eat, or the water we drink, is unknown. If this, or any of the endemics which I have mentioned, depended on the filth and dirty habits of the people, we ought to have similar complaints in Sion, or the Jew's quarter in Rome, the narrow lanes of Naples, and the stinking allies of all Italian towns and cities. But such is not the case. The Jew's quarter in Rome is the dirtiest and the healthiest spot in that famous city. The inhabitants of Fondi, Itri, and other wretched villages, in the Neapolitan dominions, are eaten up with dirt, starvation, and malaria; but no pellagra, no elephantiasis, no goitre, no cretinism is to be seen. The inevitable and the rational inference is, that each country, where peculiar or endemic maladies prevail, produces them from some hidden source, which human knowledge has not yet been able to penetrate. The general opinion among the medical men of the Milanese is, that the pellagra results from the extreme poverty and unwholesome diet of peasantry." (P. 76.)

Upon the perplexing and very important subject of *malaria*, that "dreadful scourge of Italy's fair fields," Dr. Johnson gives us much useful information. What malaria is, is not, he thinks, easily determined: but we may safely conclude that, generally speaking, it is the product of animal and vegetable decomposition, by means of heat and moisture.

"In so luxuriant a climate as that of Italy, and more especially in the southern vales and on the fertile alluvions, near the mouths of her rivers, we may well suppose that during the hot months, every spot, almost every particle of matter, teems with animal, as well as vegetable life. As the scale of existence descends, in the animal kingdom, the amazing circle of reproduction and decay is perpetually trodden by myriads of animated beings, whose ephemeral vitality has scarcely commenced before it closes again in death. The earthy tenement of the sojourner is no sooner deserted than it is resolved, by the heat and moisture of the climate, into its constituent elements, and formed without delay into other compounds. It is during this dissolution of animal and vegetable remains, preparatory to new combinations and successive reproductions, that a certain inexplicable something is extricated, which operates with such powerful and baneful influence on the functions of the human frame. Such is malaria. The materials for its generation are obvious enough in many places, as the Pontine fens, the Maremmæ, &c., but in many other places, and the Campagna among the rest, the causes of this pestiferous exhalation are more obscure. The existence of a marsh, however, is not necessary for the production of malaria. Water imbued with animal and vegetable matters may sink into the soil, and either remain there, or percolate under the surface till it finds an issue in a spring or river. This is known to be the case in numerous instances, and in almost every country. Thus, in Sicily, Dr. Irvine tells us that, ' in many of the *fiumares*, the stream disappears in the gravel, and percolates under the surface to the ocean. It is in these kinds of *fiumares* that a malaria prevails; and this probably accounts for the extrication of miasmata in many parts of the West Indies, as well as in Europe." It was too fatally ascertained by our troops in Spain and Portugal, that the dangerous season was the hot months, when the ground cracked with the heat, and permitted exhalations to issue from the moisture below the surface. We now see how it is that cultivation is no protection, in some places, from malaria. Thus, on the sloping and level ground near the lake of Bolsena, where the ruins of St. Lorenzo attest the pestiferous exhalations from a highly cultivated soil, we can easily imagine that the waters from the neighbouring hills, impregnated with vegeto-animal matters, may percolate under the surface of the soil, in their way to the lake, and, in July and August, may be exhaled in the form of malaria. The following is an illustration. "Thus (says Irvine) some places in Sicily, though on very high ground, are sickly, as Ibesso or Gesso, about eight miles from Messina, situated upon some secondary mountains lying on the side of the primitive ridge, which run northwards towards the Faro. It stands very high: but still there is some higher ground at some miles distance. Water is scarce here, and there is nothing like a marsh.'

" But eminences in Italy, and in other countries where the summer heat is tropical, are exposed to another source of malaria besides the exhalations from' their own soil, viz: the miasmata that are wafted on winds passing over malarious districts, and impinging against the first high grounds they meet. It is notorious that the heights at some distance from marshes are often more insalubrious than the immediate vicinity of the marshes themselves. Thus travellers and sojourners in Italy, during the summer, are not exempt from danger by keeping to elevated positions. They may escape fevers and agues, the more prominent features of malarious maladies, but they run the risk of imbibing the taint of a poison which will evince its deleterious influence for years afterwards, in forms anomalous and unsuspected, but more destructive of health and happiness than the undisguised attacks of remittent and intermittent fevers. The surface of the globe can hardly present a country better calculated for the generation of malaria, and for the production of those conditions of the atmosphere which give activity to the poison, than the south-west coast of Italy. Her sloping valleys are all furrowed by the beds of mountain torrents, which play the same part as the fiumari in Sicily, and form innumerable sources of malaria. Her suns are nearly as hot in summer and autumn, as those which glow over the coast of Coromandel. The south-west, on which all the principal cities stand, is exposed to the choking sirocco, which, coming parched and burning from the Lybian sands, drinks up immense quantities of aqueous vapour from the Mediterranean sea, before it rolls in volumes of boiling steam over the face of fair Italy. Under the enervating influence of these siroccos, the human frame languishes, the vital energies are depressed, the pores are opened, and the susceptibility to malarious impressions is fearfully augmented. And not to miasmal exhalations only is this susceptibility increased, but to all the dire consequences of those great and sudden atmospherical vicissitudes produced by the chilling tramontanes from the Alps, or Appennines, and the furnace blasts from Barbary. Hence it is that the inhabitants of this boasted climate are more afflicted with rheumatisms, pleurisies, and pulmonary inflammations than the inhabitants of Great Britain, in addition to the large class of diseases induced by a tropical heat, and an invisible but deadly malaria." (P. 120.)

The effects of malaria are well known to be dreadfully severe: Dr. Johnson gives a sketch of the most prominent of them. The range of disorders produced by the poison of malaria is very extensive.

"The jaundiced complexion, the tumid abdomen, the stunted growth, the stupid countenance, the shortened life, attest that habitual exposure to malaria saps the energy of every bodily and mental function and drags its victims to an early grave. A moment's reflection must shew us that fever and ague, two of the most prominent features of the malarious influence, are as a drop of water in the ocean, when compared with the other less obtrusive, but more dangerous, maladies that silently but effectually disorganize the vital structures of the human fabric, under the operation of this deleterious and invisible poison." (P. 124.)

Yet the English traveller or sojourner in Italy thinks, if he escapes the malaria fever of July and August, he has nothing more to dread, but every thing to enjoy, throughout the year.

"Fatal mistake! The foundation of chronic maladies, that render life miserable for years, is every summer laid in hundreds of our countrymen, who wander about beneath the azure skies of Italy. They bring home with them a poison circulating in their veins, which ultimately tells on the constitution, and assumes all the forms of Proteus, harassing its victim with a thousand anomalous and indescribable feelings of wretchedness, inexplicable alike to himself and his physician. It is the atttibute, the character, of all malarious disorders to be slow in their developement, when the poison is inhaled in a dilute state, or only for a short time. Many of our soldiers did not feel the effects of the Walcheren malaria till months, or even years, after that fatal expedition. So our countrymen in India often go on for years in tolerable health, after exposure to a malaria, before the noxious agent shews itself in the disturbance of certain functions of the body. The same thing is seen even in England, though on a smaller scale. Those who inhabit marshy or damp situations become, sooner or later, affected with some of the Proteiform maladies engendered by malaria, though they are seldom understood, unless they happen to take on a regular aguish character." (P. 125.)

If we compare the range of human existence, as founded on the decrement of human life in Italy and England, we find that, in Rome, a twenty-fifth part of the population pays the debt of nature annually: in Naples, a twenty-eighth part dies; in London, only one in forty, and in England generally only one in sixty, falls beneath the scythe of time or the ravages of disease.* The "value of life," in the technical language of the insurance companies, is nearly double in England what it is in Naples.

"In adducing these facts, I do not mean to deny that, in particular disorders, or in certain states of the human constitution, a specific period of the year in Italy may not conduce to the restoration of health, or at all events to the prolongation of life. But this I firmly believe, that every year's residence in Italy not only curtails the duration of life in the proportion above mentioned, but sows the seeds of such an additional crop of bodily (perhaps mental) infirmities, as will embitter the remaining years of existence, in fully as great a ratio as they diminish them." (P. 126.)

As malarious exhalations act strongly, and very injuriously, on the digestive organs and the nervous system, the range of their influence is wide, beyond all calculation. One general character, however, appertains to all the disorders connected with a malarious origin, *periodicity*, or remissions and exasperations.

"Whenever this phenomenon (periodicity) shews itself, malaria should be suspected; and those countries or localities which are infested by this destructive agent should be avoided. The misfortune is that, both in England and Italy, the poison is often introduced into the constitution, in doses so minute, that no immediate effect is produced, especially while the excitement of novelty, and the exhilaration of travelling last. When these are over, the penalty of residence in malarious countries will, sooner or later, be paid; though, even then, by sufferings, which are rarely traced or attributed to their real origin. Their nature being mistaken, the treatment is ineffectual, and health is sacrificed!" (P. 127.)

Once more we are compelled to part company from the

* See Hawkins's Statistics, 1829.

author, and to refer our readers to the work itself for an account of his route through Rome, Naples, Pompeii, Genoa, &c. In such a course, ample materials are afforded for amusement and philosophic reflection, and Dr. Johnson has most skilfully "cultivated the fruit it offers."

The third part of the work describes the influence, moral, physical, and medicinal, of an Italian climate and Italian residence, in sickness and in health. From the relative situation of the Alps, the Apennines, and the sands of Africa, it may be said that almost every breeze in Italy comes over a volcano or an iceberg, and, consequently, we are alternately scorched by the one, and frozen by the other. The *variability* of climate differs much in England and Italy: in the former, the changes, barometrical, thermometrical, and hygrometical, are very *frequent*, but their *range* is very *limited*. In the latter, it is the reverse: the transitions are *not* very frequent, but, when they do occur, the range is often most extensive.

"Now the frequency of alternations in England, and the moderate range of these alternations, are the very circumstances which render them comparatively innocuous. We have cloud and sunshine, heat and cold, winds and calms, drought and rain, twenty times in one day at home; but the British constitution becomes inured to them, and safely so, from the rapidity of their recurrence and the limitation of their range. Nay, this perpetual scene of atmospheric vicissitudes not only steels us against their effects, but proves an unceasing stimulus to activity of body and mind, and, consequently, to vigour of constitution." (P. 257.)

Sir Humphry Davy, in his "Consolations of Travel," has given a similar opinion respecting the climate of England, which he considers to be "most fitted for activity of mind, and the least suited to repose." That the climate of Italy is much less favorable to the health and longevity of men than that of England has been unequivocally proved.*

"But the question may be raised, is the climate of Italy injurious to strangers who are only temporary residents in that country? This question, I conceive, hinges essentially on the extent of the temporary residence. If the sojourn continues during a whole year; that is, throughout the entire range of the seasons, I think injury, of greater or less amount, will be sustained by the constitution, not perhaps in the shape of immediate or actual illness, but in the reception of those germs of disease which are afterwards to take on activity and growth.

"The opinions which have been broached or entertained by medical writers, both in this and other countries, respecting the medicinal effects of certain places of resort, should be received with

* Hawkins's Statistics.

caution, if not with distrust. If half the diseases which are said to be cured by Cheltenham, Bath, Harrogate, and other places, were really arrested in their course, we cannot help wondering that any one should be suffered to die in these islands. But however delicious may be the climates of Rome, Naples, Pisa, &c. we find that the greater number of medical practitioners, as well as English families, leave these interesting spots in the summer, and place the Alps between them and fair Italy. Of those who remain on the Italian side of these mountains, all who can afford the time or expense, remove to certain localities, where the air is more cool, and the malaria less prevalent than in the cities and on the plains.

"There are some, whose circumstances or inclinations induce them to remain permanently in Florence, Rome, and Naples. Very few of these last fail to exhibit the marks of a deleterious climate in their countenances. Even those who enjoy the advantage of migration to Switzerland during the malarious season, acknowledge that they begin to feel the depressive and injurious effects of the Roman air from the time they cross the Apennines on their return to the Eternal City." (P. 264.)

That people in health may wander through Italy, in safety, between September and June, the author admits; nor does he think it probable that even a sedentary residence in that classic land would be injurious during the winter, with common precaution against the climate.

"If this view of the subject be correct, it abridges not the rational pleasure of a tour through the most renowned country on the surface of this globe, a tour capable of affording instruction as well as pleasure, and, what, perhaps, is superior to both, a conviction, on returning, that England, with all its faults and imperfections, has little cause to envy the nectarious grapes, the savoury olives, the cloudless skies, or the scented gales of Italy." (P. 265.)

On the very important subject of the influence of an Italian climate on pulmonary consumption, the sum total of our knowledge appears to stand thus:

"1. In delicate health, without any proof of organic changes in the lungs, in what is called a 'tendency to pulmonary affection,' a journey to Italy, and a winter's residence there, (under strict caution,) offer probabilities of an amelioration of health: 2. In cases where there is a suspicion or certainty of tubercles in the lungs, not softened down or attended with purulent expectoration, an Italian climate may do some good, and may do much harm, the chances being pretty nearly balanced: 3. Where tuberculous matter appears in the expectoration, and where the stethoscope indicates that a considerable portion of the lungs is unfitted for respiration, a southern climate is more likely to accelerate than retard the fatal event, and takes away the few chances that remain of final recovery.

"If this be a correct estimate (it is at least an honest one) of the influence of an Italian climate on constitutions, disposed to, or affected by, pulmonary consumption, it shews that medical men incur a fearful responsibility in proposing to the parents and friends of invalids, a measure which is fraught with danger, involved in uncertainty, and too often attended by the most destructive sacrifices of the feelings, as well as the finances, of the parties concerned!" (P. 270.)

But there is a large class of complaints which *resemble* consumption, and which, Dr. J. has no doubt, contribute much to the reputation of southern climates for the cure of that terrible scourge. These are bronchial affections, viz. chronic inflammation or irritation of the mucous membrane of the lungs. People affected with nervous complaints, or disorders of the digestive organs, may generally derive benefit from a *journey* to Rome, "but it is very doubtful if a residence there would be productive of substantial good." Dr. Johnson concludes his discussion of this subject by remarking,

"That the dyspeptic, nervous, or hypochondriacal invalid, cannot adopt a more salutary maxim or principle in Italy, than that which the home secretary has laid down for the guidance of the new police in England, 'Keep moving.'" (P. 279.)

We are conscious that the sketch we have given of this work does not introduce Dr. Johnson so favorably to the public as its merits deserve. We do not, however, accuse ourselves either of negligence or injustice: he has, as we have before stated, introduced various discussions, with which we, as medical journalists, could not meddle, although they contain abundant proofs of his originality of mind, acute observation, and fluent and impressive writing.

